# Study on the design, synthesis, and activity of anti-tumor staple peptides targeting MDM2/MDMX

**DOI:** 10.3389/fchem.2024.1403473

**Published:** 2024-06-07

**Authors:** Jian Yang, Xiufei Liao, Damin Hu, Jinqiu Mo, Xiurong Gao, Hongli Liao

**Affiliations:** ^1^ The Third Affiliated Hospital of Chengdu Medical College (Chengdu Pidu District People’s Hospital), Chengdu, China; ^2^ School of Medicine, Tarim University, China; ^3^ School of Pharmacy, Chengdu Medical College, Chengdu, China

**Keywords:** staple peptides, double-targeted, anti-tumor, MDM2, MDMX

## Abstract

Staple peptides, which have a significantly enhanced pharmacological profile, are promising therapeutic molecules due to their remarkable resistance to proteolysis and cell-penetrating properties. In this study, we designed and synthesized a series of PMI-M3-based dual-targeting MDM2/MDMX staple peptides and compared them with straight-chain peptides. The staple peptide SM3-4 screened in the study induced apoptosis of tumor cells *in vitro* at low μM concentrations, and the helix was significantly increased. Studies have shown that the enhancement of staple activity is related to the increase in helicity, and SM3-4 provides an effective research basis for dual-targeted anti-tumor staple peptides.

## 1 Introduction

In contrast to cancer chemotherapy, which not only causes serious side effects in patients but may also lead to cancer recurrence due to drug-induced DNA damage and mutations (Stine et al., 2022; Thomas et al., 2022), targeted molecular therapy is superior because it targets tumor cells while targeting specific proteins or signaling pathways that promote or inhibit tumorigenesis without affecting normal cells (Balon et al., 2022; Vendrely et al., 2022).

Tumor suppressor protein p53 is one of the potential molecular targets in anticancer therapy; it is a transcription factor that plays an important role in cell differentiation, autophagy, senescence, angiogenesis, programmed cell death, and metabolism, and the increase in p53 expression will inhibit cancer cell proliferation. p53 is inhibited by the overexpression of the endogenous regulator MDM2/MDMX. Many studies have shown that restoring endogenous p53 activity can inhibit tumor growth *in vitro* and *in vivo*. By targeting MDM2/MDMX and activating the p53 protein, MDM2 and MDMX can bind to the N-terminal reverse transcription domain (TAD) of p53 through the N-terminal domain of their amino acid residues, directly inhibiting the reverse transcriptional activity of p53 and promoting the MDM2/MDMX-mediated degradation of p53. Research has focused on the design of different types of MDM2/MDMX antagonists to activate p53, and some MDM2 inhibitors have entered clinical trials targeting MDM2 to activate the p53 protein and inhibit tumor growth (Li et al., 2017; Daniele et al., 2021).

Studies have shown that MDM2 and MDMX synergistically inhibit p53 activity and cellular stability in some tumors, requiring bispecific antagonists to achieve robust and sustained p53 activation for optimal therapeutic outcomes. Therefore, targeting the tumor suppressor p53 and its negative regulator MDM2/MDMX to activate p53 *in vitro* and *in vivo* provides a feasible therapeutic strategy for cancer therapy (Soares et al., 2017; Niu et al., 2018).

Previously, the research group used phage display technology to identify a potent p53 peptide activator PMI (TSFAEYWNLLSP), which has an affinity for MDM2 and MDMX in the low-nanomolar concentration range (Zhan and Lu, 2011; Brown et al., 2013; Wade et al., 2013). Functional analysis of peptide analogs with more than 100 PMIs using surface plasmon resonance and fluorescence polarization resulted in a dodecade peptide PMI-M3 (LTFLEYWAQLMQ) that binds well to MDM2 and MDMX (Zhan et al., 2012a; Zhan et al., 2012b; Li et al., 2021).

**FIGURE 1:**
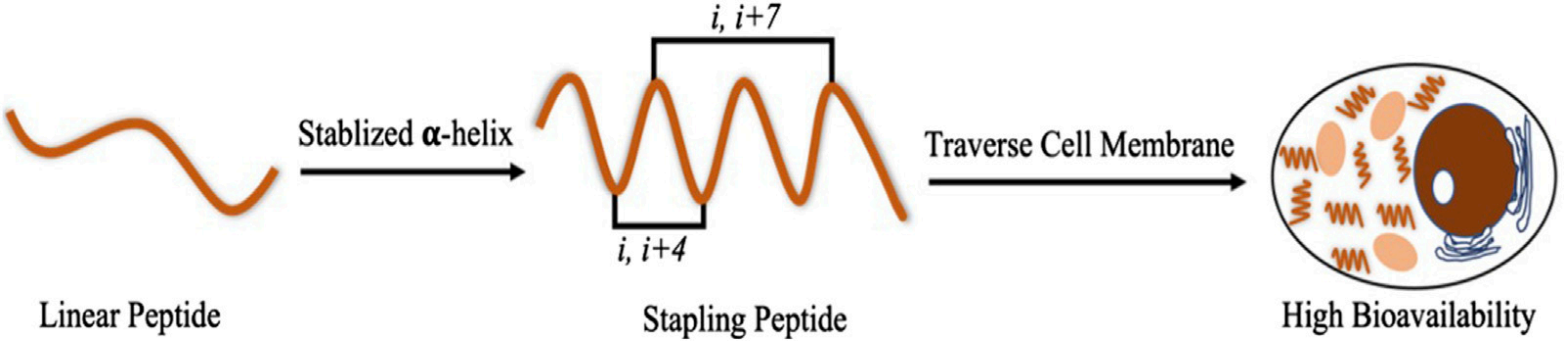
Stabilized α-helical structures of staple peptides and their advantages.

The purpose of this study was to synthesize a series of PMI-M3 dual-targeting MDM2/MDMX-based staple peptides by solid-phase synthesis, purify and lyophilize the pure products, and then measure the anti-tumor cell activity. Compared with straight-chain peptides, the α staple peptide can enhance the interaction between polypeptide molecules and proteins (Leverson, 2016; Anananuchatkul et al., 2020; Islam et al., 2022), and the staple peptide is more permeable to cell membranes, has better stability, and is expected to have higher pharmacological activity than small-molecule drugs and protein drugs (Figure 1).

## 2 Experimental section

### 2.1 Materials

Rink amide resin (0.3 mmol g^−1^ loading) was purchased from Tianjin Nankai Hecheng Science & Technology Co. Ltd (Tianjin, China). Fmoc-protected amino acids were purchased from Shanghai GL Biochem Ltd (Shanghai, China). Phenol, DIC, and oxyma TIPS were obtained from Adamas-beta (Shanghai, China). Grubbs first-generation catalyst was purchased from Sigma (United States). DMF and DCM were purchased from Wohua Chemical Co. Ltd (Shanghai, China). Ethyl ether, NMP, TFA, piperidine, and other common reagents were purchased from Sinopharm Chemical Reagent Co. Ltd (Shanghai, China). The HCT116 p53^+/+^, HCT116 p53^−/−^, U87 MG, and U251 cells were obtained from the Shanghai Cellular Institute of the Chinese Academy of Sciences (Shanghai, China).

### 2.2 Methods

#### 2.2.1 Synthesis of the staple peptides

**FIGURE 2:**
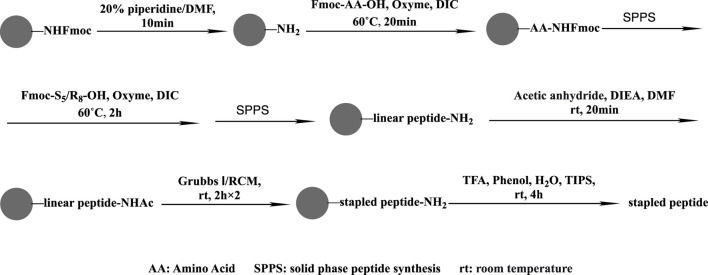
Staple peptide synthesis route.

Rink amide resin (0.66 g; 0.3 mmol g^−1^ loading capacity) was swollen with DCM (5 mL) for 20 min and then treated with 20% piperidine in DMF (10 mL), followed by washing with DMF (five times), DCM (five times), and DMF (five times). Fmoc-AA-OH (1 mmol), oxyma (1 mmol), DIC (1 mmol), and NMP (6 mL) were mixed and added to the resin to complete the coupling of the first amino acid for 20 min at 60°C. The steps of deprotection, coupling, and washing were repeated. Two equivalents of the unnatural amino acid were used during the 2-h coupling of S_5_/R_8_. Next, the Ac reside was introduced to the resin using 6 mL of pyridine and acetic anhydride (v/v = 1:1) at an ambient temperature for 20 min. Two 2-h RCM reactions in 1,2-dichloroethane at ambient temperature were carried out using Grubbs first-generation catalyst at a concentration of 10 mmol/L. Then, 87.5% TFA, 5% H_2_O, 5% phenol, and 2.5% TIPS were used for 4 h at room temperature to disconnect the crude staple peptide from the resin. After filtration, the solution was precipitated with 40 mL of ice ether, centrifuged, and blown with nitrogen to obtain the staple peptide (Figure 2).

#### 2.2.2 Preparative and analytical HPLC and mass spectrometry

The staple peptides were purified by RP-HPLC, and HPLC analysis confirmed that all purified peptides were more than 95% pure.

Using preparative HPLC (LC-1 SHIMADZU) and a YMC-Pack ODS-AQ Column (250 mm × 20 mm; IDS: 10 µm, 12 nm), the desired peptides were purified with a flow rate of 15 mL/min monitored at 214 nm.

HR-Q-TOF-MS was performed using an Agilent 6538 UHD Accurate-Mass Q-TOF mass spectrometer.

#### 2.2.3 CD spectroscopy

The staple peptides were soluble in PB with a pH of 7.2 at a concentration of 10–50 mmol/L. CD experiments were performed using a Jasco J-715 Spectropolarimeter at ambient temperature. A quartz cuvette with a 1-mm path length was used for the spectrum collection (wavelength, 185–255 nm; step resolution, 0.0001 mm; speed, 1.2 μm/h; accumulations, 6; and bandwidth, 0.0001 mm). We calculated the helicity of each peptide using Eq.

#### 2.2.4 Cytotoxicity assay

Human colon cancer cell lines HCT116 p53^+/+^, HCT116 p53^−/−^, U87 MG, and U251 were maintained in McCoy’s 5A Medium supplemented with 10% heat-inactivated FCS and 1% penicillin–streptomycin at 37°C, 5% CO_2_, and under complete humidification conditions. The cells (3×10^3^ cells/well) were inoculated in 96-well plates and treated with different concentrations of polypeptides for 72 h. The absorbance at 450 nm was then measured using the CCK-8 Kit, and the percentage of cell survival was calculated based on the ratio of the sample well to the reference well A450.

#### 2.2.5 Apoptosis assay

Fluorescent probes were used to detect normal viable cells, apoptotic cells, and necrotic cells by double labeling of cells, and the morphological characteristics of the apoptotic cells were explored. A Petri dish with 8–90% confluence was taken from the incubator. Tumor cells were collected, and the cells were counted under the microscope through the cell slide. A cell suspension of the appropriate concentration was prepared. Different concentrations of peptide samples were added according to the experimental group. Three compound wells were set up in each group; the control group was set up without peptides, and the blank group was set up without cells. Then, 300 μL of Binding Buffer was added after 24 h of dosing, and 2 μL of Annexin V was added to each tube, pipetted to mix it well, and then incubated at room temperature in the dark for 15 min. Then, 2 μL of PI solution was added before placing it on the machine, and one tube without dye was used as a negative control. On-machine assays were performed with flow cytometry. The results showed that the UL region showed cell necrosis, the LL region showed cell survival, the L region showed early apoptosis, and the UR region showed late apoptosis. Total apoptosis = LR region + UR region.

### 2.3 Results

#### 2.3.1 Structural characterization of the SM series of targeted anti-tumor peptides

The targeted MDM2/MDMX staple peptides SM3-1, SM3-2, SM3-3, SM3-4, SM3-5, and SM3-8 (Table 1) were prepared according to the solid-phase synthesis route in this study, and the results of the structural characterization of the staple peptides were verified by mass spectrometry to match their theoretical molecular weights (Table 2).

**TABLE 1:** SM staple peptide sequence.

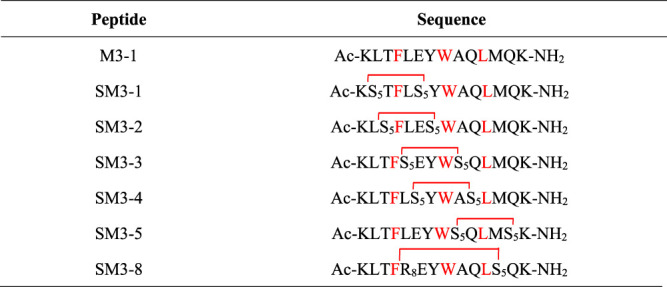	

**TABLE 2:** Structural characterization results of staple peptides obtained by Q-TOF/MS.

Name	Molecular formula	Molecular weight (g/mol)	Ion (m/z)
SM3-1	C_91_H_138_N_20_O_19_S	1,848.2860	[M+2H]^2+^ = 925.0178; [M+3H]^3+^ = 617.0148
SM3-2	C_89_H_140_N_20_O_19_S	1,826.2800	[M+2H]^2+^ = 914.0153; [M+3H]^3+^ = 609.6803
SM3-3	C_93_H_140_N_20_O_21_S	1,906.3220	[M+2H]^2+^ = 954.0242; [M+3H]^3+^ = 636.3520
SM3-4	C_92_H_141_N_19_O_18_S	1,833.3150	[M+2H]^2+^ = 917.5285; [M+3H]^3+^ = 612.0221
SM3-5	C_94_H_143_N_19_O_20_S	1,891.3510	[M+2H]^2+^ = 946.5461; [M+3H]^3+^ = 631.3679
SM3-8	C_94_H_142_N_20_O_21_	1,888.2890	[M+2H]^2+^ = 945.0452; [M+3H]^3+^ = 630.3657

#### 2.3.2 Circular dichroism characterization

**FIGURE 3:**
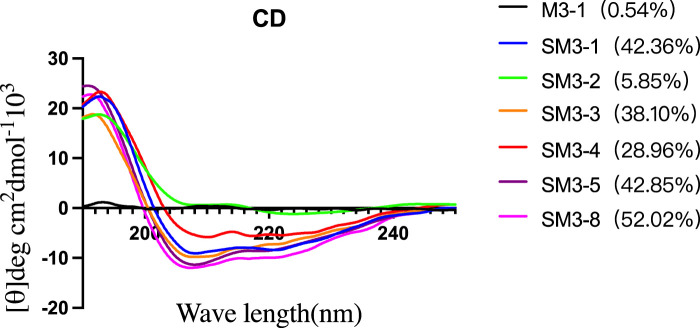
Circular dichroism and degree of helicity of peptides.

In this study, circular dichroism was used to characterize the secondary structure of the synthesized peptides and determine their helicity. As shown in Figure 3, compared with the linear peptide M3-1, the helicity of the staple peptide SM series was significantly increased, and the helicity of SM3-1 increased to 42.36%. SM3-2 helicity increased to 5.85%; SM3-3 helicity increased to 38.10%; SM3-4 helicity increased to 28.96%; SM3-5 helicity increased to 42.85%; and SM3-8 helicity increased to 52.02%.

#### 2.3.3 Targeting anti-tumor peptide SM series cytotoxicity

**FIGURE 4:**
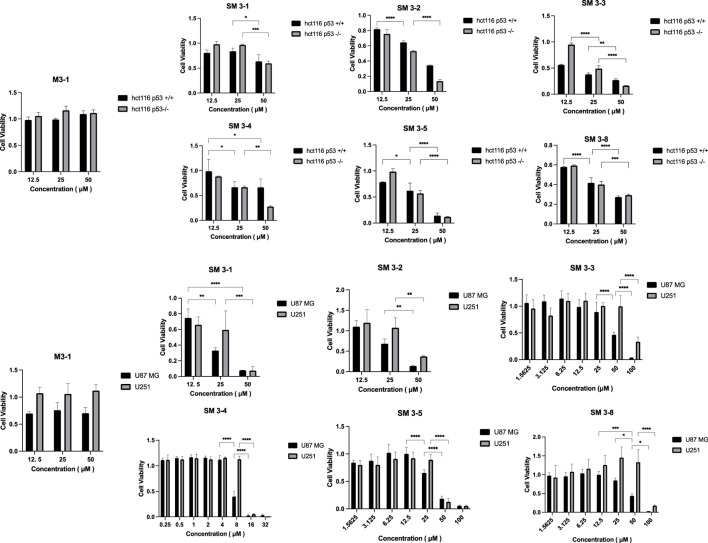
Peptide cell-viability test results. Note: Tumor cells were treated with staple peptides for 2 or 7 days and tested for cytotoxicity using the CCK-8 reagent. The results are presented as the mean ± standard deviation (‾x ± SD). *: *p* < 0.05.

In this study, tumor cells HCT116 p53^+/+^, HCT116 p53^−/−^, U87 MG, and U251 were tested for cytotoxicity of the synthesized peptides, and the results of the cell viability of the peptides were verified (Figure 4). The staple peptide IC_50_ results were calculated (Table 3).

**TABLE 3:** IC_50_ value of staple peptides.

Name	HCT116 p53^+/+^	HCT116 p53^−/−^	U87 MG	U251
M3-1	∼	∼	∼	∼
SM3-1	118.50	54.81	19.02	22.64
SM3-2	34.36	24.04	30.7	48.67
SM3-3	15.62	25.81	47.51	93.01
SM3-4	77.48	33.19	7.839	14.74
SM3-5	27.27	27.4	31.18	35.91
SM3-8	17.55	17.88	45.02	94.16

Note: Tumor cells were treated with staple peptides for 2 or 7 days and tested for cytotoxicity using the CCK-8 reagent. Units of data: µM. ∼: very wide.

Compared to the linear peptide M3-1, SM3-1, SM3-2, SM3-3, SM3-4, SM3-5, and SM3-8 exhibited *in vitro* anti-tumor activity at a concentration of 25 μM. The analysis of the activity of U87 MG tumor cells showed that the inhibitory activity of all staple peptides was significantly better than that of straight-chain peptides, and SM3-3 and SM3-8 had *in vitro* anti-tumor activity at 50 μM, and SM3-1, SM3-2, and SM3-5 had *in vitro* anti-tumor activity at 25 μM. The results of the activity of U251 tumor cells showed that the inhibitory activity of all staple peptides was significantly better than that of straight-chain peptides, and SM3-1, SM3-2, and SM3-5 had *in vitro* anti-tumor activity at 50 μM, and SM3-3 and SM3-8 had anti-tumor activity *in vitro* at 100 μM. Among them, the most active SM3-4 had a minimum inhibitory concentration of 8 μM for tumor cell U87 MG and a minimum inhibitory concentration of 16 μM for tumor cell U251.

#### 2.3.4 Targeting anti-tumor peptide SM series apoptosis

**FIGURE 5:**
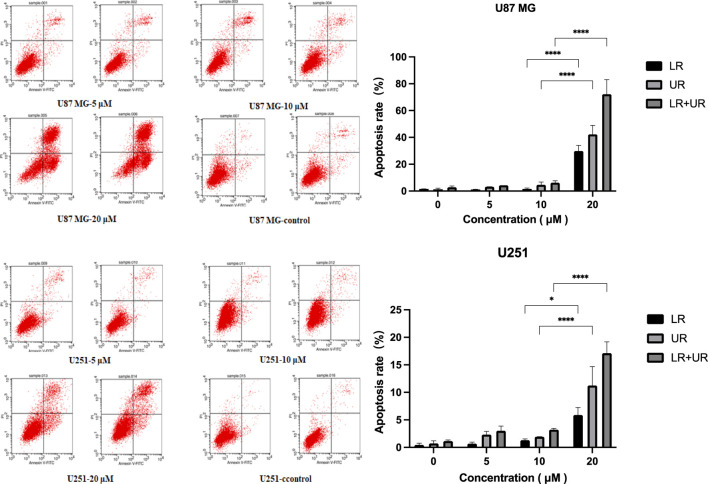
SM3-4 cell apoptosis assay results. Note: U87 MG and U251 cells were treated with SM3-4 for 36 h to detect normal viable and apoptotic cells using Annexin V. U87 MG at a polypeptide concentration of 20 μM; LR: apoptotic cells, 29.91%; UR: dead cells, 42.285%. U251 at a polypeptide concentration of 20 μM; LR: apoptotic cells, 5.885%; UR: dead cells, 11.23%. The results are presented as the mean ± standard deviation (‾x ± SD). *: *p* < 0.05.

Tumor cells U87 MG and U251 were dual labeled using fluorescent probes to detect normal viable cells and apoptotic cells, and the apoptosis test results of SM3-4 cells are shown in Figure 5.

The results showed that SM3-4 had a significant apoptotic effect on tumor cells U87 MG and U251, and with the increase in concentrations of 5 μM, 10 μM, and 20 μM, the number of apoptotic cells in the LR region (early-apoptosis cells) and UR region (late-apoptosis cells) was significantly increased, with 20 μM showing the highest increase.

## 3 Conclusion

In this study, the amino resin was used as the solid-phase support material, and DIC–oxyme was used as the reaction coupling system. The target staple peptides were obtained by Fmoc solid-phase synthesis, and the S5/R8 amino acid side chain groups were linked to polypeptide cyclization by the olefin cross-metathesis reaction. Six N-terminal-acetylated target MDM2/MDMX staple peptides were successfully obtained.

The results of circular dichroism showed that the helicity of the staple peptide formed by the linking and cyclization of S5/R8 amino acid side chain groups was significantly higher than that of the control straight-chain peptide. Tumor cells HCT116 p53^+/+^, HCT116 p53−/−, U87, and U251 were selected for cell viability experiments, and the results of cell activity experiments showed that the tumor suppressive activity of the staple peptide was significantly higher than that of the template straight-chain peptide, indicating that the enhanced activity of the staple peptide was related to the increase in helicity. The anti-proliferative activity of staple peptides SM3-2, SM3-4, and SM3-5 was significantly improved. Among them, the staple peptide SM3-4 had the strongest anti-proliferative activity.

## Data Availability

The datasets presented in this study can be found in online repositories. The names of the repository/repositories and accession number(s) can be found in the article/Supplementary Material.
