# Supplementary material for: Study on the design, synthesis, and activity of anti-tumor staple peptides targeting MDM2/MDMX
Source: Front Chem. 2024 Jun 7;12:1403473. doi: 10.3389/fchem.2024.1403473 (PMC11190158; doi:10.3389/fchem.2024.1403473)
Supplement: Supplementary file 1 [file DataSheet1.ZIP › SM project raw data/Apoptosis/2021.03.04 Apoptotic sequence.docx]

2021.03.04凋亡顺序

1-U 87 MG 5 μM 上

2- U 87 MG 5 μM 下

3- U 87 MG 10 μM 上

4- U 87 MG 10 μM 下

5- U 87 MG 20 μM 上

6- U 87 MG 20 μM 下

7- U 87 MG 对照 上

8- U 87 MG 对照 下

9-U 251 5 μM 上

10-U 251 5 μM 下

11-U 251 10 μM 上

12-U 251 10 μM 下

13-U 251 20 μM 上

14-U 251 10 μM 下

15-U 251 对照 上

16-U 251 对照 下
