# Supplementary figures and images for: Study on the design, synthesis, and activity of anti-tumor staple peptides targeting MDM2/MDMX
Source: Front Chem. 2024 Jun 7;12:1403473. doi: 10.3389/fchem.2024.1403473 (PMC11190158; doi:10.3389/fchem.2024.1403473)

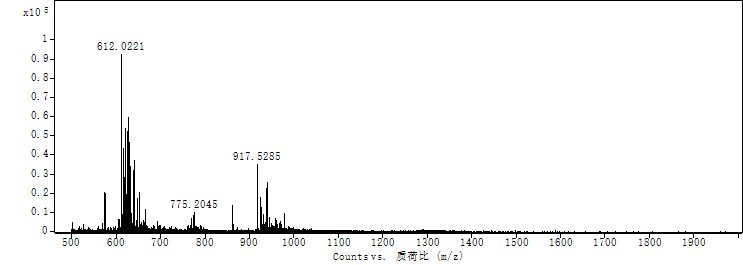

Supplement: Supplementary file 1 [file DataSheet1.ZIP › SM project raw data/SM series staple peptide high-resolution mass spectrometry results/SM3-4/1.bmp]

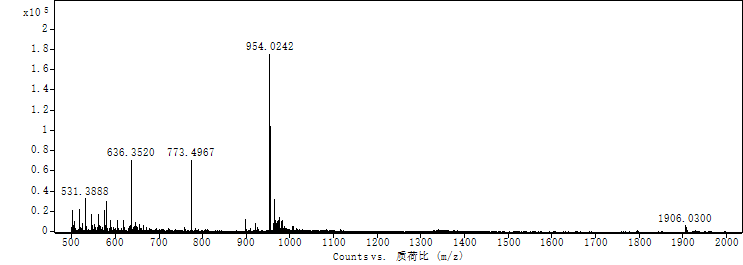

Supplement: Supplementary file 1 [file DataSheet1.ZIP › SM project raw data/SM series staple peptide high-resolution mass spectrometry results/SM3-3/1.bmp]

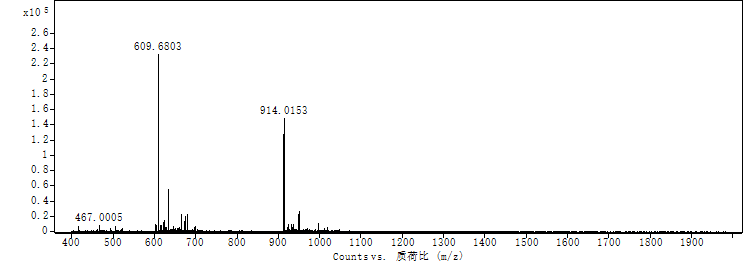

Supplement: Supplementary file 1 [file DataSheet1.ZIP › SM project raw data/SM series staple peptide high-resolution mass spectrometry results/SM3-2/1.bmp]

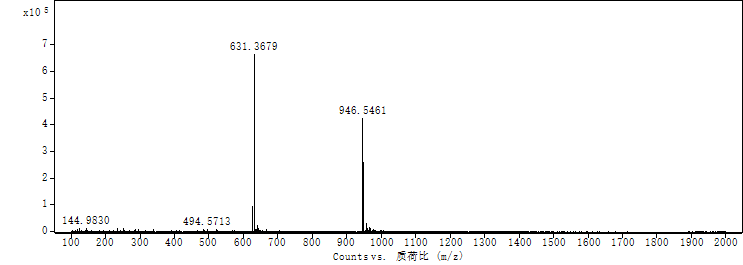

Supplement: Supplementary file 1 [file DataSheet1.ZIP › SM project raw data/SM series staple peptide high-resolution mass spectrometry results/SM3-5/1.bmp]

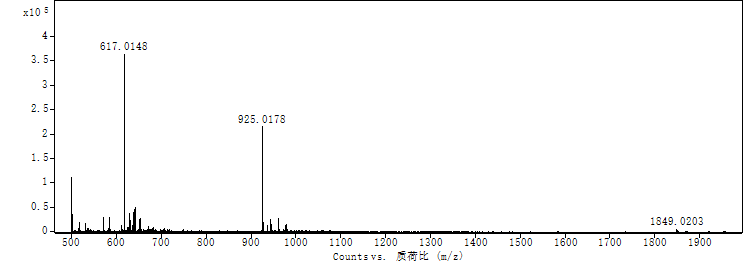

Supplement: Supplementary file 1 [file DataSheet1.ZIP › SM project raw data/SM series staple peptide high-resolution mass spectrometry results/SM3-1/1.bmp]

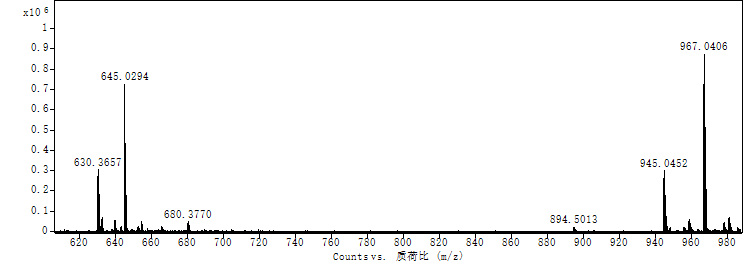

Supplement: Supplementary file 1 [file DataSheet1.ZIP › SM project raw data/SM series staple peptide high-resolution mass spectrometry results/SM3-8/1.bmp]
